# Resting HRV Sample Entropy Predicts the Magnitude of Post-Exercise Vagal Withdrawal in Young Adults

**DOI:** 10.3390/medicina61101766

**Published:** 2025-09-30

**Authors:** Valters Vegelis, Ieva Anna Miezaja, Indra Mikelsone, Antra Jurka

**Affiliations:** Department of Human Physiology and Biochemistry, Riga Stradiņš University, LV-1007 Riga, Latvia

**Keywords:** exercise, heart rate variability, autonomic nervous system, sustained attention, equivalence testing, IPAQ, PSQI

## Abstract

*Background and Objectives:* Acute exercise lowers vagal HRV, yet it is unclear who will show the largest drop and whether simple questionnaires can identify them. To test whether resting HRV complexity (Sample Entropy) predicts the magnitude of acute vagal withdrawal and whether this physiology-based marker has greater practical utility than self-report activity/sleep measures for screening and recovery decisions. *Materials and Methods*: In a single-arm pre–post experimental study, twenty-nine students (20.4 ± 0.5 y; 13 males, 16 females) completed one morning visit (08:00–12:00 h). After a 2 min resting ECG and a Sustained Attention to Response Task (SART), participants cycled 15 min at 0.85 × (220 − age) bpm following a 5 min 25 W warm-up. HRV was re-recorded within ~2 min and SART ~5 min post exercise. The IPAQ defined low/medium/high activity tertiles. Correlations related baseline measures to change scores. *Results*: RMSSD decreased by −12.93 ms [−25.71, −2.03] (*p* = 0.003, r = 0.60) and SDNN by −14.91 ms [−22.30, 7.66] (*p* = 0.011, r = 0.51). Reaction time shortened slightly (−8.77 ms [−59.33, 30.40], *p* = 0.35). Activity tertiles did not differ in ΔRMSSD, ΔSDNN, or ΔRT (all *p* > 0.10). Sample Entropy predicted autonomic change (ΔRMSSD r = 0.43, *p* = 0.034; ΔSDNN r = 0.59, *p* = 0.002), whereas the PSQI and IPAQ did not. Equivalence tests showed non-significant tertile differences were not within our predefined equivalence bounds. *Conclusions*: Individuals with more complex resting HRV were more likely to show a larger immediate vagal withdrawal after moderate cycling. Questionnaires did not identify these responders. Non-linear HRV may aid practical screening/monitoring, whereas self-reports alone appear insufficient. Generalizability is limited by the homogeneous young adult sample.

## 1. Introduction

Acute aerobic exercise is known to reduce the influence of the vagus nerve on heart rate variability (HRV) indices such as RMSSD and SDNN during the initial minutes of recovery, while producing only small, short-term improvements in cognitive performance (e.g., faster reaction time) [1,2]. Yet most studies emphasize average effects and under-report when, relative to exercise cessation, HRV is sampled: a methodological choice that can materially change what is observed [3]. Early (0–2 min) sampling captures steep vagal withdrawal, while later windows show partial rebound [4,5]. Because HRV reactivity varies with age, sex, fitness/stimulants, and sleep activity patterns that differ across cultures, our findings from a homogeneous young adult student cohort should be generalized with caution and verified in more diverse samples [6,7,8].

While linear HRV indices (e.g., RMSSD, SDNN) quantify the amount of beat-to-beat variation (short-term vagal modulation), non-linear complexity metrics (e.g., Sample Entropy, DFA-α1) capture the temporal structure and unpredictability of RR dynamics—features linked to adaptability and regulatory flexibility. Because complexity reflects how flexibly the system reorganizes over time, it offers information about regulatory adaptability that amplitude-based metrics may miss [9,10].

Most recent studies use non-linear HRV to characterize current states—for example, how complexity shifts within graded exercise or across recovery stages—rather than to predict a future individual response. In contrast, we test whether a baseline complexity marker (Sample Entropy) can prospectively forecast the magnitude of post-exercise vagal withdrawal in each participant. In addition to these well-described linear HRV metrics, non-linear “complexity” measures (e.g., Sample Entropy, DFA α1) are gaining popularity because they reflect the richness and adaptability of autonomic control rather than its amplitude alone [9,11,12]. However, contemporary papers have mostly used complexity to describe responses across states rather than to predict the magnitude of an individual’s future response [10,11,13]. Far fewer studies have asked whether baseline complexity can predict how strongly an individual’s vagal tone will change after exercise [9].

Researchers often choose low-cost questionnaires, such as the International Physical Activity Questionnaire (IPAQ) and Pittsburgh Sleep Quality Index (PSQI), to evaluate habitual behavior. However, both tools show only modest concurrent validity with objective measures and possess considerable measurement noise [14,15,16,17]. Evidence in comparable young adult samples is mixed: some studies find low association with accelerometry (over-/under-reporting of intensity domains), while others report acceptable reliability/structure in college cohorts, especially in online formats. Meta-analytic work generally rates IPAQ concurrent validity as modest versus devices [14,18,19]. The PSQI is widely used, yet its factor structure and measurement invariance vary across populations. Some student samples show acceptable reliability/validity online, whereas recent appraisals question a single-factor model and highlight population-specific structure [20,21]. A PSQI score greater than 5 is the conventional “poor sleep” cut-off, yet its physiological relevance may vary across samples [22,23]. Whether these self-reports meaningfully forecast acute autonomic or cognitive responses remains unclear.

Growing interest in “individual responders vs. non-responders” has highlighted that apparent heterogeneity can reflect measurement error, sampling windows, or analytic choices rather than biology [24,25,26]. Contemporary methodological papers therefore urge the use of equivalence/TOST procedures or Bayesian evidence to distinguish between “no effect” and “evidence for no meaningful effect” [27,28]. Visual tools that classify responders across multiple domains (e.g., autonomic and cognitive) can also clarify patterns masked by group means [24,25].

From a practical standpoint, the ability to anticipate who will show a large immediate vagal withdrawal after moderate exercise could help clinicians and coaches individualize load, cool-down duration, and recovery monitoring and flag those who may need closer follow-up. If a brief resting ECG-derived complexity marker provides this forecast, it offers a low-burden alternative to questionnaires for screening and day-to-day decision-making.

In our study, we aimed to test whether resting HRV complexity (Sample Entropy) predicts the magnitude of acute vagal withdrawal response to a standardized 15 min moderate-to-vigorous submaximal cycling bout and whether this physiology-based marker outperforms habitual physical activity (IPAQ) and sleep quality (PSQI) scores. We further quantified inter-individual variability with a responder taxonomy (autonomic vs. cognitive) and applied equivalence testing to judge whether non-significant tertile differences were practically trivial. This multi-pronged approach aims to deliver both mechanistic insight (explaining why some individuals show larger autonomic shifts) and practical guidance for screening/monitoring in exercise settings.

We hypothesized that higher resting HRV complexity (Sample Entropy) would prospectively predict a larger immediate decrease in vagal indices (ΔRMSSD, ΔSDNN) within ~2 min after the cycling bout. We further expected that the IPAQ and PSQI would not explain additional variance in these autonomic changes beyond Sample Entropy and that habitual activity tertiles would show no meaningful differences in ΔRMSSD/ΔSDNN (i.e., within a ±5 ms smallest worthwhile difference).

## 2. Materials and Methods

This was a single-arm, pre–post experimental study in which each participant served as their own control. Thirty-five students were invited; thirty consented; one failed inclusion criteria, yielding twenty-nine healthy university students (13 males, 16 females; age 20.4 ± 0.5 y; BMI 22.17 ± 2.81 kg·m^−2^) who completed a single ~60 min morning laboratory session. Testing was scheduled between 08:00 and 12:00 h to minimize circadian variability in HRV indices and sustained attention. Inclusion criteria were age 20–21 years, no diagnosed cardiovascular/metabolic/neurological/psychiatric disorders, no regular medication affecting autonomic function, and the ability to perform moderate cycling. Exclusion criteria were acute illness or injury; failure to abstain ≥ 6 h from caffeine, nicotine, alcohol, or strenuous exercise; and poor-quality ECG/HRV recordings (>5% artefacts after correction).

### 2.1. Study Variables

Sleep quality was assessed with the Pittsburgh Sleep Quality Index (PSQI). Habitual physical activity was captured with the International Physical Activity Questionnaire (IPAQ; total and domain MET-min·week^−1^). Resting HRV was derived from a 2 min ECG and included time–domain indices (RMSSD, SDNN) and a non-linear index (Sample Entropy).

We selected RMSSD as the primary time–domain measure because it reflects short-latency vagal modulation, is robust to respiratory rate, and shows excellent short-term reliability even in 1–2 min segments, which is important for early post-exercise sampling [29,30]. SDNN was included to complement RMSSD vagal focus. To assess regulatory complexity, we used Sample Entropy, which quantifies the temporal structure and unpredictability of RR dynamics rather than amplitude alone and thus informs on adaptive capacity [31]. We prioritized Sample Entropy over Approximate Entropy because it excludes self-matches, reduces bias and record-length dependence, and performs more reliably on short, noisy RR series—well suited to 2 min post-exercise windows [31].

Cognitive performance was measured using the Sustained Attention to Response Task (SART) built into PsyToolkit. The SART probes sustained attention and response inhibition. It is widely used for reaction-time paradigms and is sensitive to arousal, which shifts with transient vagal withdrawal after exercise [32,33,34]. Thus, it provides a tightly timed cognitive readout aligned with the autonomic change we measured. Thirty digits (0–9) were presented in random order; participants clicked for every number except “3”. Both reaction time (ms) and accuracy (% correct withholds/commission errors) were recorded. Change scores (Δ) were calculated as post–pre for all HRV and cognitive outcomes.

### 2.2. Experimental Protocol

After arrival and completing the questionnaire, participants rested quietly. Quiet-rest standardization: seated upright with back support, feet flat, legs uncrossed, hands on thighs, eyes open, spontaneous breathing, and no talking, movement, or device usage. We allowed 2–3 min for stabilization and repeated segments if artefact criteria were not met. A 2 min artefact-free ECG was recorded at 1000 Hz (PowerLab/ADInstruments, LabChart 8 HRV). R-peaks were detected in LabChart and RR intervals were exported to Kubios HRV for preprocessing. Kubios automatic beat-correction was applied (threshold-based + default settings). Every segment was then visually inspected to confirm correct R-peak placement and the absence of movement/EMG noise or baseline wander. A priori quality criteria required ≤ 5% corrected beats per 2 min segment, and recordings exceeding this threshold were repeated immediately. The pre-exercise SART followed the resting HRV recording.

Participants then completed a standardized warm-up (5 min at 25 W on an Ergoselect 200 ergometer). Over the next 1–2 min, workload was increased and then adjusted to maintain 0.85 × (220 − age) for the remainder of the 15 min bout. This target yields a vigorous yet submaximal workload without maximal testing and is a pragmatic age-predicted HR max approach for young adults [35]. Cadence was 70–80 rpm. Heart rate (HR) was monitored continuously from the ECG, using a 15–30 s running average to guide changes. If averaged HR fell ≥ 5 bpm below target, resistance was increased by 10–15 W. If averaged HR rose ≥ 5 bpm above target, resistance was decreased by 10–15 W. Adjustments were limited to once per minute to allow stabilization, and HR was capped at ≤90% of age-predicted HR max. Target HR was maintained in real time. Mean exercise HR was 169.38 ± 3.17 bpm.

**Figure 1:**
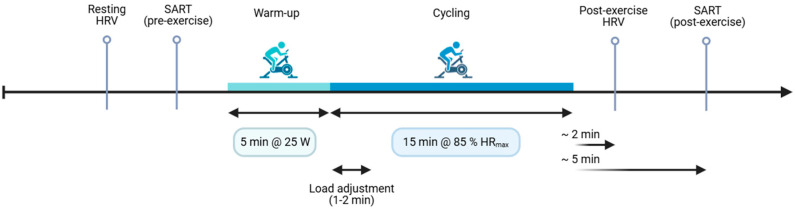
Experimental timeline. After questionnaires, a 2 min resting ECG was recorded for HRV analysis, followed immediately by the pre-exercise SART. Participants then completed a 5 min warm-up at 25 W, after which workload was adjusted over ~1–2 min to reach and maintain 85% of age-predicted HR max for 15 min of cycling. Within ~2 min of exercise cessation, a second 2 min ECG was obtained (post-exercise HRV), followed by the post-exercise SART (~5 min). The entire laboratory visit was conducted in the morning (8:00–12:00) and lasted approximately 60 min.

Within ~2 min of exercise cessation, we recorded a second 2 min ECG under identical conditions, then administered the post-exercise SART (~5 min post exercise). The 2 min post-exercise window was chosen a priori to capture early recovery when vagal withdrawal is maximal and because 1–3 min segments provide reliable short-term HRV indices [29,30]. All testing occurred in a quiet, temperature-controlled room, and the visit lasted ~60 min (Figure 1).

### 2.3. Statistical Analysis

Analyses were performed in SPSS v29. Distributions were checked with the Shapiro–Wilk test. Because most variables were not normally distributed, we report median [IQR]. Pre–post differences were evaluated with non-parametric paired tests (Wilcoxon signed-rank or sign test where ties dominated). Effect sizes for paired tests are reported as r = |Z|/√N.

Habitual activity tertiles (low/medium/high) were created by tertiling total IPAQ scores. We used tertiles because activity was skewed and the sample modest, giving balanced rank-based groups that are robust to outliers and suitable for non-parametric tests. We also analyzed activity continuously (Spearman) to limit information loss from categorization. Between-group differences in change scores used the Kruskal–Wallis test with η^2^ as effect size.

Associations between baseline measures and Δ variables used Spearman’s ρ (non-normal distributions, some ordinal predictors, and monotonic, possibly non-linear, relations). Responders were classified with sign-based thresholds (ΔRMSSD < 0, ΔRT < 0) and summarized as proportions.

To distinguish “no effect” from “no meaningful effect”, we ran two one-sided tests (TOST) against ±5 ms bounds for ΔRMSSD/ΔSDNN and ±10 ms for ΔRT, using Welch 90% CIs. Failure to fall entirely within bounds indicated non-equivalence. Sensitivity of equivalence decisions was checked by repeating TOST with ±3/±7 ms (HRV) and ±5/±15 ms (RT) bounds and by expressing bounds as ±0.2 × baseline SD. For interpretation, we defined effect-size thresholds: small/medium/large = 0.10/0.30/0.50 for r and ρ; 0.01/0.06/0.14 for η^2^. Two-tailed α = 0.05 without multiplicity adjustment, equivalence decisions were based on CI containment and TOST *p*-values.

We complemented TOST by mapping the same equivalence bounds to a region of practical equivalence (ROPE) (HRV: ±5 ms; RT: ±10 ms) and computing Bayes factors for the tertile contrasts; posterior mass within the ROPE and Bayes factors are reported in the Supplement.

### 2.4. Ethics Approval and Consent to Participate

Following verbal and written explanation of the study, written informed consent was obtained from all participants. This study was approved by the Riga Stradiņš University Research Ethics Committee (2-PĒK-4/522/2024) and carried out according to the Declaration of Helsinki. Confidentiality was safeguarded by assigning coded IDs, keeping the re-identification key in a separate encrypted file (PI-only access), and storing de-identified analysis datasets on encrypted, password-protected university servers. Only aggregated results are reported. Safety was ensured through pre-exercise screening, a standard warm-up/cool-down, continuous ECG/heart rate monitoring with predefined stop criteria, and supervision by trained staff with an AED available. No adverse events occurred.

## 3. Results

### 3.1. Baseline Parameters

Before exercise, all distributions were non-normal (Shapiro–Wilk *p* < 0.05), so values are reported as the median [IQR]. The PSQI was 10 [9–11] (28/29 > 5). Total IPAQ activity was 2890 [1332–4127] MET-min·week^−1^ (vigorous 462 [297–693], moderate 720 [200–2400], walking 480 [0–1920]). Resting HRV demonstrated an RMSSD of 45.61 ms [36.01–79.20] and SDNN of 54.12 ms [38.58–79.56]; an HF power of 910.22 ms^2^ [478.25–2018.55]; and a Sample Entropy of 1.64 [1.49–1.73]. Resting heart rate was 73.5 bpm [65.3–76.3]. Exploratory sex comparisons showed no differences at baseline RMSSD (*p* = 0.63), SDNN (*p* = 0.75), and SampEn (*p* = 0.62). Pre-exercise SART reaction time (RT) was 160.0 ms [125.1–181.8] and accuracy was 92% [88–96]. Activity tertiles contained 10 low, 9 medium, and 10 high participants. Complete pairs were available for HRV 25/29 and RT 27/29.

### 3.2. Autonomic Responses to Exercise

**Figure 2:**
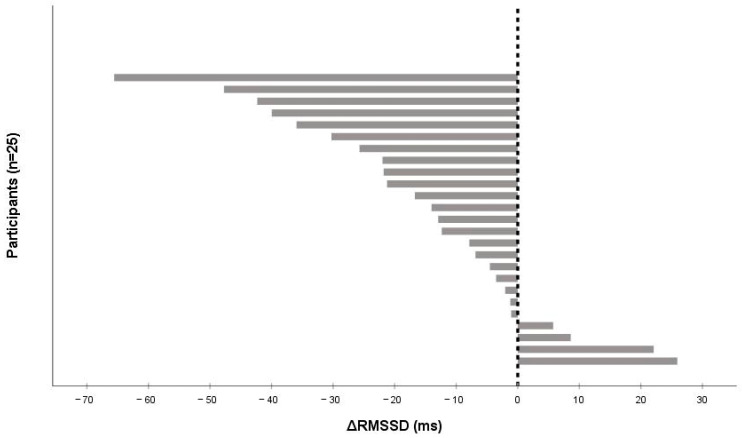
Individual changes in RMSSD (ΔRMSSD) from pre- to post-exercise after a 15 min moderate-to-vigorous submaximal cycling bout (n = 25). Bars are sorted from the largest decrease to the largest increase; negative values indicate vagal (parasympathetic) withdrawal. The vertical line denotes zero change.

After the 15 min cycling bout, parasympathetic HRV indices fell noticeably: RMSSD by −12.93 ms [−25.71 to −2.03] (*p* = 0.003, r = 0.60) and SDNN by −14.91 ms [−22.30 to 7.66] (*p* = 0.011, r = 0.51). Sample Entropy fell slightly (Δ −0.142 [−0.332 to −0.029]). Heart rate rose to 83.5 bpm [70.5 to 89.5]. Change in HRV indices did not vary between males and females for ΔRMSSD (*p* = 0.63) and ΔSDNN (*p* = 0.71). Individual autonomic responses varied widely (ΔRMSSD range −66.0 to +26 ms; Figure 2). From a practical perspective, these median drops correspond to ~28% (RMSSD) and ~27% (SDNN) relative to baseline medians (RMSSD 45.61 ms; SDNN 54.12 ms) and exceed our ±5 ms smallest worthwhile difference (SWD), indicating a meaningful immediate vagal withdrawal.

The wide spread in ΔRMSSD (−66 to +26 ms) likely reflects individual differences in vagal ‘reserve’/baseline complexity, the size of the exercise-induced HR rise (ΔHR), and early recovery dynamics (0–2 min is non-stationary and sensitive to spontaneous breathing). Because HRV scales inversely with heart rate, participants with larger post-exercise HR elevations tend to show larger RMSSD drops; accordingly, we provide a supplementary regression (ΔRMSSD ~ ΔHR + Sample Entropy_pre + mean RR_pre) to illustrate how these factors account for between-person variance (Table S3).

### 3.3. Cognitive Changes

Cognitive effects were small. RT shortened by −8.77 ms ([−59.33 to 30.40]; *p* = 0.35, r = 0.18), and accuracy did not change (Δ 0% [−4 to 4], *p* = 0.82). There was no statistically significant difference in ΔRT between males and females (*p* = 0.66). Individual RMSSD responses ranged from −66 to +26 ms (Figure 2). Practically, the RT shift is ~6% of the baseline median (160 ms), suggesting limited impact on attention in this setting.

### 3.4. Habitual Activity Comparisons

**Figure 3:**
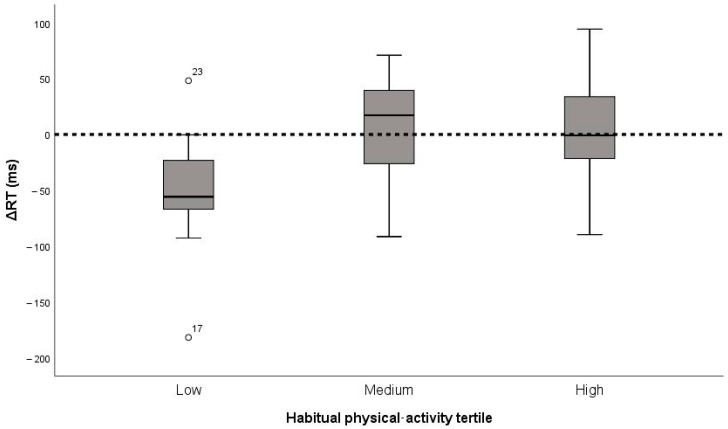
Change in reaction time (ΔRT) after the 15 min cycling bout by habitual physical activity tertile (low, medium, high; n = 29). Boxes show medians and inter-quartile ranges; whiskers represent 1.5 × IQR. The horizontal line denotes zero change (no effect).

Change scores did not differ significantly between low (n = 10), medium (n = 9), and high (n = 10) activity tertiles. ΔRMSSD medians were −21.80, −7.86, and −12.93 ms (χ^2^ = 1.35, *p* = 0.51, η^2^ < 0.01, small); ΔSDNN medians were −18.07, −8.24, and −16.48 ms (χ^2^ = 2.92, *p* = 0.23, η^2^ < 0.04, small); ΔRT medians were −55.9, +9.1, and +4.4 ms (χ^2^ = 4.48, *p* = 0.11, η^2^ < 0.10, borderline-medium). Thus, habitual activity did not materially modulate the acute responses (Figure 3).

### 3.5. Baseline Predictors (Complexity vs. Questionnaires)

**Figure 4:**
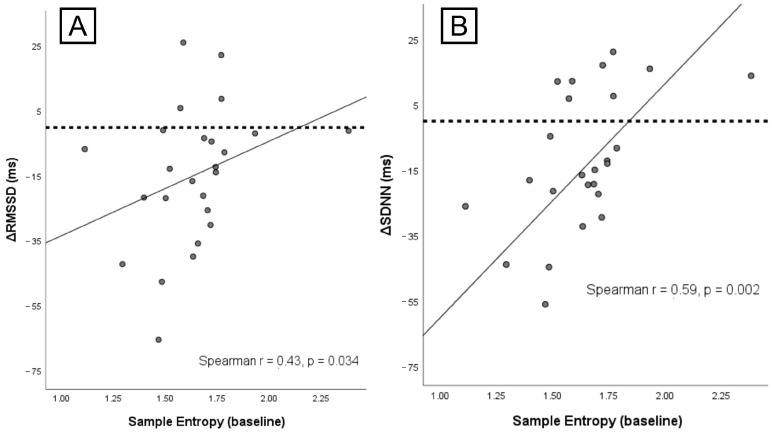
Associations between baseline HRV complexity and autonomic change. (**A**) Sample Entropy at rest vs. ΔRMSSD (r = 0.43, *p* = 0.034). (**B**) Sample Entropy at rest vs. ΔSDNN (r = 0.59, *p* = 0.002). Lines show linear fits; points are individual participants. Negative Δ values indicate larger vagal withdrawal.

Resting HRV complexity (Sample Entropy) predicted the magnitude of autonomic withdrawal: ΔRMSSD r = 0.43, *p* = 0.034; ΔSDNN r = 0.59, *p* = 0.002 (Figure 4). In contrast, PSQI showed no association with ΔRMSSD (r = −0.10, *p* = 0.65) or ΔRT (r = 0.20, *p* = 0.33). Likewise, total IPAQ score did not relate to ΔRMSSD (r = 0.24, *p* = 0.25) or ΔRT (r = 0.29, *p* = 0.14). Baseline RMSSD did not predict ΔRT (r = 0.08, *p* = 0.70). Higher resting complexity identified individuals more likely to show a meaningful immediate vagal withdrawal, while questionnaires did not offer comparable predictive value.

### 3.6. Responder Taxonomy (Autonomic–Cognitive Quadrants)

**Figure 5:**
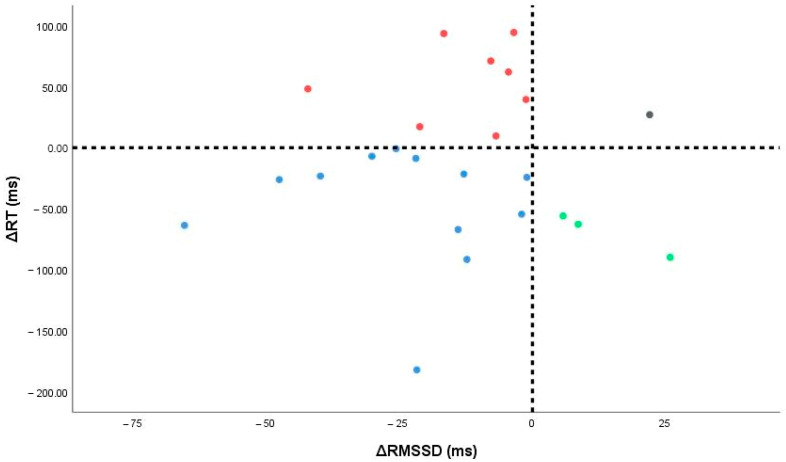
Individual changes in autonomic (ΔRMSSD) and cognitive (ΔRT) measures. Vertical and horizontal lines denote zero change; points fall into four responder quadrants (dual, autonomic-only, cognitive-only, non-responders).

Using sign thresholds (ΔRMSSD < 0 = autonomic responder; ΔRT < 0 = cognitive responder), 24 participants had complete paired data: 12/24 (50.0%) were dual responders, 8/24 (33.3%) were autonomic-only, 3/24 (12.5%) were cognitive-only, and 1/24 (4.2%) were non-responders (Figure 5). Five cases (17.2%) lacked one paired measure and were excluded.

Viewed physiologically, these quadrants may reflect distinct profiles of arousal–autonomic coupling. Dual responders likely exhibit pronounced vagal withdrawal with concurrent arousal-related speeding of responses. Autonomic-only responders show strong cardiac vagal shifts without measurable cognitive change. Cognitive-only responders may gain arousal-driven RT benefits despite modest autonomic change. Meanwhile, non-responders may reflect higher stability, ceiling effects, or measurement noise. Exploratorily, baseline complexity (Sample Entropy), which correlated with autonomic change in our data, appears a plausible marker of vagal “reserve”, helping explain why some participants show larger ΔRMSSD than others. However, categories overlapped, and numbers were small, so these physiotype labels are hypothesis-generating only and require further investigation with larger samples. See Table S1 for baseline medians [IQR] by quadrants.

### 3.7. Equivalence and Sensitivity (Habitual Activity Effects)

To test whether non-significant tertile differences were also practically trivial, we ran TOST with ±5 ms bounds for ΔRMSSD/ΔSDNN and ±10 ms for ΔRT. All Welch 90% CIs crossed these bounds (e.g., ΔRMSSD low–medium −13.2 ms [−34.2, 7.9]; low–high −16.4 ms [−36.7, 3.9]; medium–high −3.2 ms [−19.1, 12.6]). Analogous patterns were seen for ΔSDNN and ΔRT (e.g., ΔRT low–high −57.9 ms [−107.5, −8.3]). No comparison met equivalence (all TOST *p* > 0.05), indicating we cannot assert practical equivalence with current precision. Observed differences were small to modest (η^2^ ≤ 0.10). A brief Bayesian sensitivity using the same ROPE bounds yielded convergent inferences (posterior mass largely within ROPE; Bayes factors did not support meaningful between-tertile differences). See Table S3 for pairwise contrasts and CIs.

## 4. Discussion

Our central finding is that resting HRV complexity (Sample Entropy, SampEn), rather than self-reported activity or sleep, predicts the size of acute vagal withdrawal after a brief moderate-to-vigorous cycling bout. This extends work showing non-linear HRV indices capture autonomic regulation but are rarely used to predict individual response magnitude [9,36]. Higher SampEn likely indexes a more adaptable control system (“physiological complexity”) that can mount a larger, rapid reconfiguration to load [9,37]. In contrast, questionnaires (IPAQ, PSQI) carry measurement noise and week physiological linkage, which explains their limited predictive value here [14,19,21].

Physiologically, higher resting Sample Entropy likely reflects a control system with richer multiscale feedback (central autonomic network, baroreflex, respiratory–cardiac coupling) and wider reconfiguration of vagal outflow when the system is more flexible. In contrast, lower entropy indicates more predictable, constrained rhythms—often dominated by rigid breathing–heart rate coupling—so the available dynamic range for rapid vagal withdrawal is smaller.

Mechanistically, higher SampEn may index greater “autonomic reserve”—a control system capable of faster, broader parasympathetic adjustments and baroreflex engagement during early recovery—while amplitude metrics, such as RMSSD, reflect the degree of variability present, rather than how flexibly it reorganizes under load [9,36,38]. This helps explain why SampEn, but not IPAQ/PSQI, predicted the magnitude of vagal withdrawal in our young, relatively homogenous cohort [14,19,21].

We sampled HRV within ~2 min post exercise, a window often avoided for non-stationarity yet rich in autonomic information. As expected, RMSSD and SDNN fell (medians −13 and −15 ms), while individual changes varied widely (–66 to +26 ms). This dispersion mirrors reports that recovery kinetics are highly individual and depend on intensity and sampling window [4,5,38]. Recent reviews urge reporting distributions, not just means, during early recovery because RR dynamics are especially informative then [25,38]. Three factors plausibly drive the wide individual spread we observed: first—the inverse scaling of short-term HRV with heart rate (larger post-exercise HR compresses RMSSD); second—differences in baseline control architecture captured by SampEn; third—unpaced breathing variability in early recovery, which adds scatter to time–domain indices.

The small, non-significant RT improvement (–9 ms, ~6%) aligns with Bayesian/meta-analytic evidence that acute cognitive gains are modest and short-lived (~40 min, g~0.1) [1,2,39]. Joint plotting of ΔRMSSD and ΔRT revealed autonomic-only and dual responders, implying partial decoupling between vagal withdrawal and attentional speeding [24,40]. Timing likely matters: executive benefits depend on when testing occurs relative to exercise offset [1,2].

Because null results can be misread, we complemented Kruskal–Wallis tests with equivalence testing (TOST). CIs routinely crossed conservative bounds (±5 ms HRV; ±10 ms RT), so we could not claim equivalence despite non-significance [27,28]. Practically, this means group differences by IPAQ tertiles were small and imprecisely estimated, not “proven the same”. Reducing error with objective sleep/activity and larger samples should sharpen inferences.

A “sleep × activity” interaction may also explain the small “non-responder” group. Higher activity could yield larger vagal withdrawal only when sleep is adequate, while poor or fragmented sleep might blunt reactivity even in active individuals [6,7,8]. Our poor-sleep skew and modest N limited power for interaction tests, and self-report noise likely attenuated affects. Future studies should model continuous interactions, pair questionnaires with actigraphy/wearables, and use multilevel designs across repeated sessions.

Measurement timing is crucial for interpreting complexity. The first 0–2 min include rapid vagal reactivation, breathing shifts, and elevated HR that compress variance and can depress entropy if protocols vary. We recommend fixed 2 min windows, consistent posture and breathing instructions, and reporting mean HR/RR and entropy parameters (m, r). Where possible, include timing sensitivity (0–2 vs. 3–5 min) to show robustness.

These findings fit a personalized prescription lens. Short RR capturing is now feasible on wearables or chest-strap ECG with good agreement to clinical ECG when artefact control is robust [41]. Complexity metrics are also moving into practice: DFA-α1 helps delineate intensity domains and aerobic thresholds in real time [10,25]. A pre-session 2 min SampEn could therefore flag “large withdrawal” individuals who might need longer cool-downs, early HRV spot-checks (0–10 min), or delayed cognitively demanding tasks, while in-session DFA-α1 still guides intensity [24,42,43].

For implementation, embed a 2 min seated RR capture before sessions and interpret SampEn against personal baseline. In clinics or rehab, a low or unusually depressed SampEn (large withdrawal) suggests ≥5–10 min cool-down, early post-exercise HRV check, and a 15–20 min delay before attention-heavy tasks. In team or fitness settings, adjust intensity density and recovery on low-SampEn days. Keep decisions probabilistic, combining SampEn with context (sleep, illness, heat), and favor ECG/chest-strap or validated devices. Also, standardize entropy parameters across platforms.

Limitations of our study include a small, age-homogenous sample with high poor-sleep prevalence (range restriction), a single early post-exercise HRV window, and reliance on self-report for behavior. We also did not collect stress-biology (salivary cortisol or catecholamines) or ventilation/lactate data, which could help disambiguate autonomic withdrawal from metabolic load and stress reactivity. Finally, the absence of neurophysiological measures (e.g., EEG during SART) limited mechanistic linkage between autonomic change and cognitive cortisol. Integrating autonomic and cortical indices would strengthen interpretation of autonomic–cognition coupling. Standardizing breathing and adding multiple post-exercise windows would further stabilize complexity estimates and recovery profiles. Future work should (1) combine non-linear HRV with objective activity/sleep; (2) sample multiple recovery time points; (3) use equivalence/Bayesian approaches; (4) test whether complexity-guided screening improves outcomes.

## 5. Conclusions

Our data suggest that a resting complexity marker (Sample Entropy) more accurately anticipates the magnitude of immediate vagal withdrawal after moderate cycling than self-reported activity or sleep, supporting a low-burden, precision-style screening for tailoring cool-downs and early recovery monitoring. Practically, a 2 min pre-session ECG SampEn can support personalized cool-downs, early recovery checks, and timing of cognitively demanding tasks. While strengths include a tightly standardized early recovery protocol and convergent effect sizes, generalization beyond young adults with prevalent poor sleep remains to be established. As a concise next step, multi-session within-person validation across standard recovery windows and devices in more diverse samples will confirm generalizability and operational thresholds for field use.

## Data Availability

The datasets analyzed during the current study are available from the corresponding author on reasonable request.

## Supplementary Materials

The following supporting information can be downloaded at: https://www.mdpi.com/article/10.3390/medicina61101766/s1, Table S1. Baseline characteristics by responder quadrant. Table S2. Exploratory regression explaining individual variation in ΔRMSSD. Table S3. Pairwise tertile contrasts for change scores with equivalence tests.
