# Supplementary material for: Resting HRV Sample Entropy Predicts the Magnitude of Post-Exercise Vagal Withdrawal in Young Adults
Source: Medicina (Kaunas). 2025 Sep 30;61(10):1766. doi: 10.3390/medicina61101766 (PMC12565908; doi:10.3390/medicina61101766)
Supplement: Supplementary file 1 [file medicina-61-01766-s001.zip › medicina-3862939-supplementary.pdf]

Suplementary material

Table S1. Baseline characteristics by responder quadrant

Medians [IQR] for Sample Entropy, RMSSD, SDNN, and PSQI by quadrant defined from sign-based change thresholds (ΔRMSSD < 0 = autonomic responder; ΔRT < 0 = cognitive responder). Values are descriptive/exploratory; no between-group inference was performed. n indicates the number of participants with complete paired data contributing to each quadrant.

Quadrant	n	SampEn_pre	RMSSD_pre (ms)	SDNN_pre (ms)	PSQI
Dual responders	12	1.58 [1.49, 1.72]	60.12 [35.89, 82.78]	53.33 [40.50, 82.13]	10.00 [9.75, 11.00]
Autonomic -only	8	1.68 [1.55, 1.74]	41.23 [35.34, 52.60]	51.96 [35.71, 60.84]	9.00 [8.00, 9.25]
Cognitive-only	3	1.59 [1.58, 1.68]	48.33 [33.48, 78.12]	53.24 [38.73, 72.83]	11.00 [8.50, 13.50]
Non-responders	1	1.77 [1.77, 1.77]	38.68 [38.68, 38.68]	35.00 [35.00, 35.00]	11.00 [11.00, 11.00]

Table S2. Exploratory regression explaining individual variation in ΔRMSSD

Linear model with ΔRMSSD (post − pre, ms) as the dependent variable and ΔHR (post − pre, bpm), resting Sample Entropy (SampEn_pre), and resting mean RR interval (meanRR_pre, ms) as predictors. Estimates are from OLS; 95% CIs are percentile bootstrap intervals (2,000 resamples). n = 25.

Term	Estimate	Std. Error	t	p-value	Boot 95% CI lower	Boot 95% CI upper
Intercept	−49.972	30.758	−1.625	0.119	−112.111	2.130
ΔHR (bpm)	−1.162	0.368	−3.160	0.0047	−1.895	−0.095
SampEn_pre	19.763	16.050	1.231	0.232	−3.696	48.756
meanRR_pre (ms)	0.021	0.025	0.843	0.409	−0.026	0.062

Model fit: $R^2 = 0.397$, adjusted $R^2 = 0.311$.

Table S3. Pairwise tertile contrasts for change scores with equivalence tests

Pairwise mean differences (g1–g2) with Welch 90% CIs and TOST p-values against pre-specified equivalence bounds (HRV ±5 ms; RT ±10 ms) for change scores: ΔRMSSD, ΔSDNN, ΔRT. Group sizes correspond to participants with non-missing change scores in each tertile. No comparison met the equivalence criterion (all TOST p > 0.05).

Outcome	Contrast	n(g1)	n(g2)	Mean diff (g1–g2)	90% CI lower	90% CI upper	Welch df	TOST bounds	TOST p
ΔRMSSD	Low – Medium	7	9	−13.174	−34.194	7.847	11.52	±5 ms	0.750
ΔRMSSD	Low – High	7	9	−16.383	−36.652	3.886	10.36	±5 ms	0.833
ΔRMSSD	Medium – High	9	9	−3.209	−19.061	12.643	15.66	±5 ms	0.423
ΔSDNN	Low – Medium	7	9	−18.814	−38.777	1.150	13.14	±5 ms	0.879
ΔSDNN	Low – High	7	9	−12.286	−30.416	5.843	10.85	±5 ms	0.757
ΔSDNN	Medium – High	9	9	6.527	−9.902	22.956	14.74	±5 ms	0.564
ΔRT	Low – Medium	9	8	−53.575	−105.493	−1.656	15.00	±10 ms	0.919
ΔRT	Low – High	9	10	−57.897	−107.501	−8.294	16.34	±10 ms	0.944
ΔRT	Medium – High	8	10	−4.323	−52.680	44.035	15.29	±10 ms	0.420
